# Opioid suppression of conditioned anticipatory brain responses to breathlessness

**DOI:** 10.1016/j.neuroimage.2017.01.005

**Published:** 2017-04-15

**Authors:** Anja Hayen, Vishvarani Wanigasekera, Olivia K. Faull, Stewart F. Campbell, Payashi S. Garry, Simon J.M. Raby, Josephine Robertson, Ruth Webster, Richard G. Wise, Mari Herigstad, Kyle T.S. Pattinson

**Affiliations:** a Nuffield Department of Clinical Neurosciences (NDCN), University of Oxford, Oxford, UK; b Department of Psychology, University of Reading, Reading, UK; c Nuffield Department of Anaesthetics, Oxford University Hospitals NHS Foundation Trust, Oxford, UK; d Cardiff University Brain Research Imaging Centre, School of Psychology, Cardiff University, Cardiff, UK; e Department of Infection, Immunity and Cardiovascular Disease, University of Sheffield, Sheffield, UK

**Keywords:** Breathlessness, Opioid, Anticipation, Conditioning, FMRI, Breathing

## Abstract

Opioid painkillers are a promising treatment for chronic breathlessness, but are associated with potentially fatal side effects. In the treatment of breathlessness, their mechanisms of action are unclear. A better understanding might help to identify safer alternatives. Learned associations between previously neutral stimuli (e.g. stairs) and repeated breathlessness induce an anticipatory threat response that may worsen breathlessness, contributing to the downward spiral of decline seen in clinical populations. As opioids are known to influence associative learning, we hypothesized that they may interfere with the brain processes underlying a conditioned anticipatory response to breathlessness in relevant brain areas, including the amygdala and the hippocampus.

Healthy volunteers viewed visual cues (neutral stimuli) immediately before induction of experimental breathlessness with inspiratory resistive loading. Thus, an association was formed between the cue and breathlessness. Subsequently, this paradigm was repeated in two identical neuroimaging sessions with intravenous infusions of either low-dose remifentanil (0.7 ng/ml target-controlled infusion) or saline (randomised).

During saline infusion, breathlessness anticipation activated the right anterior insula and the adjacent operculum. Breathlessness was associated with activity in a network including the insula, operculum, dorsolateral prefrontal cortex, anterior cingulate cortex and the primary sensory and motor cortices.

Remifentanil reduced breathlessness unpleasantness but not breathlessness intensity. Remifentanil depressed anticipatory activity in the amygdala and the hippocampus that correlated with reductions in breathlessness unpleasantness. During breathlessness, remifentanil decreased activity in the anterior insula, anterior cingulate cortex and sensory motor cortices. Remifentanil-induced reduction in breathlessness unpleasantness was associated with increased activity in the rostral anterior cingulate cortex and nucleus accumbens, components of the endogenous opioid system known to decrease the perception of aversive stimuli.

These findings suggest that in addition to effects on brainstem respiratory control, opioids palliate breathlessness through an interplay of altered associative learning mechanisms. These mechanisms provide potential targets for novel ways to develop and assess treatments for chronic breathlessness.

## Highlights

• The mechanisms of how low-dose opioids relieve breathlessness are unknown.
• We tested whether low-dose opioids affect conditioned anticipation and perception of breathlessness.
• Low-dose opioids reduced unpleasantness, but not intensity of breathlessness.
• Reduced breathlessness unpleasantness was associated with activation of the endogenous opioid system.
• Breathlessness relief was predicted by decreased anticipatory activity in amygdala/hippocampus.

## Introduction

Breathlessness debilitates millions of people with cardiorespiratory disease, terminal cancer and neuromuscular disorders. Chronic breathlessness often correlates poorly with objective measures of disease severity (Hayen et al., 2013, Herigstad et al., 2011, Lansing et al., 2009). This poor correlation between breathlessness and disease markers might be explained by interindividual variability in brain processing of respiratory sensations, hence the justification for neuroimaging studies. Breathlessness is considered a multidimensional symptom, including sensory components of 'work of breathing', affective and emotional components of breathlessness sensations (Hayen et al., 2013, Lansing et al., 2009) compounded by the various psychological processes associated with an individual's suffering (Hayen et al., 2013, Oxley and Macnaughton, 2016).

There has been growing interest in the use of low-dose opioids for the treatment of chronic breathlessness (Ekstrom et al., 2015, Rocker et al., 2013). The neural mechanisms of opioid-driven breathlessness relief are largely unknown (Peiffer, 2011). Opioids depress respiration by acting upon brainstem respiratory centres (Pattinson, 2008) and also act in higher brain centres involved in respiratory control (Pattinson et al., 2009). Importantly, in experimental settings low-dose opioids have been shown to differentially act on the “unpleasantness" (a component of the affective dimension) compared to the "intensity" (a component of the sensory dimension) of aversive stimuli (Price et al., 1985).

Over repeated episodes of breathlessness, associations are formed between previously neutral stimuli, e.g. a flight of stairs (conditioned stimulus [CS]) and breathlessness (unconditioned stimulus [US] (De Peuter et al., 2004)). This has two important outcomes: firstly, learnt breathlessness anticipation may increase breathlessness itself (De Peuter et al., 2005), reinforcing the CS-US pairing, and secondly, the feared activity is avoided, fuelling a downward spiral of activity avoidance, physical deconditioning and worsening breathlessness.

In this context of conditioned learning, opioids have shown profound effects on association learning and memory formation to aversive experiences (Sandkühler and Lee, 2013; Fanselow 1998; McNally 2004). Two of the key structures involved in these processes are the amygdala and hippocampus (Phelps, 2004). As these structures are rich in opioid receptors (Favaroni Mendes 2008; McGaraughty and Heinricher, 2002), we hypothesised that opioid effects on association learning with regards to breathlessness are mediated, at least in part, by the amygdala and hippocampus.

Therefore, the effect of opioids on breathlessness most likely stems from multiple actions within the central nervous system. A better understanding of these brain mechanisms could be used to develop alternative therapies with fewer safety concerns than opioids (Pattinson, 2015, Ray et al., 2016, Robertson et al., 2016, Vozoris et al., 2016). In the present study we used functional neuroimaging to identify the neural correlates of the conditioned response to breathlessness and their modulation by the mu-opioid receptor agonist remifentanil. We tested the hypothesis that in addition to direct effects on respiration, remifentanil would also act on neural mechanisms associated with conditioned learning in the amygdala and hippocampus.

## Methods

This double-blind, randomized, placebo-controlled mechanistic study investigated the neural correlates of the opioid remifentanil upon the anticipation and perception of breathlessness. An aversive delay-conditioning session was followed by two FMRI sessions (remifentanil or saline placebo, counterbalanced across participants). The sessions were performed on three consecutive days at the same time each day.

### Data acquisition

#### Participants

Data from 19 healthy participants (10 females, age 24 (±7 SD) years) was analysed in this study. Written informed consent was obtained in accordance with the Oxfordshire Research Ethics Committee. Although 29 participants originally participated, 10 were excluded for the following reasons: 2 participants exhibited vasovagal syncope during cannulation; 1 participant did not comply with study instructions; 4 participants did not learn the association between visual cues and respiratory stimuli; 3 participants were excluded because of technical difficulties with the MRI equipment. Participants were right-handed non-smokers and had no history of neurological (including painful conditions), pulmonary or cardiovascular disease, were free from acute respiratory infections and were currently not receiving any medication. Participants fasted solids for 6 h and liquids for 2 h before every session.

#### Initial session

The Center for Epidemiologic Studies Depression Scale (CES-D; (Radloff, 1977)) was used to identify (and exclude) participants with clinical depression. The trait scale of the Spielberger State-Trait Anxiety Inventory (STAI; (Spielberger, 2010)) was used to characterize general participant anxiety.

#### Breathlessness stimulus

The breathlessness stimulus used in this study was intermittent resistive inspiratory loading for 30–60 s administered via the MRI compatible breathing system illustrated in Supplementary Fig. 1. Manually operated hydraulic valves diverted inspiratory flow via one of three routes that either did not restrict breathing, or provided a mild or strong resistive load. Expiration was unrestricted via a one-way valve (Hans Rudolph, Shawnee, Kansas, USA).

**Fig. 1:**
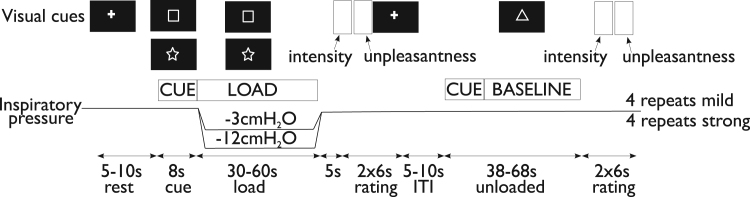
Schematic illustration of experimental session and aversive conditioning paradigm. Prior to the application of each inspiratory load, the fixation cross on the screen changed to one of three shapes, a triangle, a square and a star to signal the imminent application of a stimulus (mild inspiratory load, strong inspiratory load) for eight seconds (anticipation period). The shape remained on the screen during the application of the stimulus (stimulus period) for 30–60 s and changed back to the fixation cross when the stimulus ceased. The shapes were counterbalanced across participants. Each inspiratory load was followed by an unloaded period of between 30 and 60 s that was indicated by a third visual cue. The use of relatively long breathlessness stimuli was chosen to maximize the emotional responses associated with anticipation of breathlessness. Participants rated their respiratory intensity and unpleasantness after each stimulus. Visual stimuli were generated and presented in white on a black background using the Cogent toolbox (www.vislab.ucl.ac.uk/Cogent/) for MatLab (MathWorks Inc., Natick, MA, USA).

In an externally cued delay conditioning paradigm (Fig. 1), participants learned associations between three visual cues (conditioned stimuli, (CS), either a white square, star or triangle shape presented on a black background) and resistive inspiratory loading that was intermittently applied to induce three different respiratory sensations (unconditioned stimuli, (US), either breathlessness (strong inspiratory load, approximately −12 cm H_2_O), a mild inspiratory load (approximately −3 cm H_2_O) or no inspiratory load). The pairing between the visual cue (CS) and respiratory load (US) was maintained constant for each participant during all 3 experimental sessions, but was counterbalanced between participants. Four repeats of each of the mild load and strong load (breathlessness) and eight repeats of the unloaded condition were performed. Immediately after each inspiratory load, participants rated the intensity and unpleasantness of their breathing on a horizontal visual analogue scale (VAS) with the anchors ‘no breathlessness’ on the left and ‘severe breathlessness’ on the right for intensity ratings and ‘not unpleasant’ on the right and ‘extremely unpleasant’ on the left for unpleasantness ratings. Bond-Lader mood values of tension-relaxation, sedation-alertness, and discontentment-contentment (Bond and Lader, 1974) were obtained immediately following the breathlessness protocol using visual analogue scales (VAS) displayed on the screen and a button box.

Conscious association between CS and US was confirmed in writing and 4 participants who did not form such associations were excluded from the study. Following the breathlessness protocol, an anaesthetist performed a medical assessment; this included a 20-minute test infusion of remifentanil to ensure tolerance and safety.

The participants were allowed at least 5 min to get accustomed to the breathing system before the experiment commenced. Throughout the experiment, the partial pressure of expired carbon dioxide (P_ET_CO_2_) was maintained constant (isocapnia). This was achieved by initially increasing P_ET_CO_2_ by +0.3 kPa from baseline by adding CO_2_ to the inspired air, and then manually adjusting inspired CO_2_ as necessary (Hayen et al., 2015; Wise et al., 2007). The partial pressure of expired oxygen (P_ET_O_2_) was kept constant at 20 kPa in a similar manner.

#### Physiological recordings

Arterial oxygen saturation and heart rate were monitored continuously and non-invasive blood pressure was recorded before and after each scan (In Vivo Research, Orlando, FL, USA). P_ET_CO_2_ and P_ET_O_2_ were determined using rapidly responding gas analysers (ADinstruments ML206) and continuously displayed and recorded with a data acquisition device (PowerLab 8, AD instruments, Colorado Springs, CO, USA) connected to a laptop computer using dedicated software (Chart 5, AD instruments, Colorado Springs, CO, USA). Inspiratory gas flow was measured with a pneumotachograph (ADinstruments Spirometer FE141) and a standard flow head. Mouth pressure was measured using a Validyne pressure transducer (Validyne Engineering, 8626 Wilbur Ave Northridge, CA 91324).

#### FMRI sessions

Two FMRI sessions were performed, one on each of the two days following the initial conditioning session, and consisted of a remifentanil and saline placebo session (counterbalanced). During acquisition of the FMRI scans, participants underwent the breathlessness protocol as described above (identical to the initial session). Additional structural scans, field maps and measures of cerebral blood flow were obtained (described in detail below).

#### Drug infusion

A target-controlled infusion (TCI) pump (Graseby 3500 TCI incorporating Diprisor, SIMS Graseby Ltd, Watford, UK) delivered remifentanil (at a solution concentration of 10 μg/ml) to achieve a desired effect site concentration of 0.7 ng/ml or saline placebo (administered in the same manner) intravenously into a cannula placed in the dorsum of the left hand. The TCI pump was pre-programmed with the three-compartment pharmacokinetic model of remifentanil (Minto et al., 1997a, Minto et al., 1997b). The total duration of the infusion was 45 min, which allowed for a 10-min ramp-up period to reach the desired effect site concentration. All participants fasted for 6 h before each visit and were monitored for an hour after termination of the infusion.

Both study participants and study experimenters were blinded to the order of drug administration, with only the administering anaesthetist and the MRI scanner operator being aware of drug condition.

We used remifentanil as a model opioid because its pharmacokinetics and pharmacodynamics are ideal for a mechanistic volunteer study such as this. It is a synthetic μ-opioid agonist with a rapid onset of action, a context sensitive half-life of 3–4 min, and an elimination half-life of approximately 8–10 min. This means that the drug has extremely rapid onset and offset, and when combined with a target-controlled infusion, drug levels can be easily manipulated within a short time frame. Due to its rapid onset and offset, remifentanil needs to be administered as an intravenous infusion and when this infusion is combined with a pharmacokinetic model of its action, it is possible to manipulate plasma and effect site (brain) concentrations in a predictable and consistent manner. Using a target controlled infusion allows a constant drug effect to be maintained throughout the experiment.

In terms of volunteer safety, if adverse effects develop, the infusion can be terminated, with the knowledge that the drug will wear off within minutes. We chose to deliver an effect site concentration of 0.7 ng/ml based upon our extensive clinical and experimental experience with this drug (Bingel et al., 2011, MacIntosh et al., 2008, Mitsis et al., 2009, Pattinson et al., 2008, Wanigasekera et al., 2011, Wanigasekera et al., 2012, Wise et al., 2002, Wise et al., 2004). Although direct comparison of equivalence with other opioids (e.g. oral morphine) is difficult, we chose a dose that is at the lower end of efficacy for the treatment of acute pain but which has previously been shown to have effects on respiration (Mitsis et al., 2009) and pain suppression (Bingel et al., 2011). We would estimate that the effect site concentration of 0.7 ng/ml would represent the analgesia offered by 4–7 mg oral (or 2.0–3.5 mg intravenous) morphine used to treat low-moderate pain. Remifentanil is ultra-short acting, and this means that when given as a bolus the effects would vary within the scanning session, making it difficult to dissociate primary drug effect from secondary effects such as raised P_ET_CO_2_. For this reason, comparison with studies employing bolus doses of remifentanil (Leppa et al., 2006) is difficult.

#### MRI data acquisition

MRI data were acquired on a 3 T Siemens Trio scanner using a 32-channel head coil. A whole-brain gradient echo, echo-planar-imaging (EPI) sequence, repetition time (TR)=3000 ms, echo time (TE)=30 ms, field of view: 192×192 mm, voxel size 3×3×3 mm, 45 slices, 380 volumes) was used for functional scans. Fieldmaps were obtained using a symmetric-asymmetric spin-echo sequence before the baseline functional scans (30 ms echo time, 0.5494 ms dwell time, field of view and matrix identical to EPI) and were used to correct for magnetic field inhomogeneities. A T1-weighted structural (MPRAGE sequence, TR=1720 ms, TE=4.68 ms, flip angle 8°, voxel size 1×1×1 mm) image was acquired for functional image registration.

To account for changes in cerebral blood flow (CBF) induced by remifentanil (MacIntosh et al., 2008), a multi-inversion time pseudo-continuous arterial spin labeling sequence (multi-TI PCASL; Tis = 1.65, 1.9, 2.15, 2.4, 2.65, 2.9 s, TI cycles=6, tag duration –1.4 s, TR=4000 ms, TE=13 ms, voxel size 3.8×3.8×5.0 mm, 24 slices, 72 volumes) was run either before or after the BOLD scanning (breathlessness experiment) randomised between participants whilst the drug/placebo infusion was running. This included phase contrast measurement of blood velocity in the carotid arteries.

### Data analysis

#### Behavioural ratings

Behavioural data were analysed in SPSS (Version 21, IBM Corp., Armonk, NY). Separate 2 [drug: saline vs. remifentanil] ×2 [stimulation condition: normal breathing vs. strong loading] repeated measures ANOVAs were performed for ratings of perceived intensity and unpleasantness. The three dimensions of the Bond-Lader mood scale (alertness-sedation, relaxation-tension and contentment-discontentment) were compared in three separate 2 [drug: saline vs. remifentanil] ×2 [time: during and 15 min after infusion] repeated measures ANOVAs using Bonferroni adjustments within tests. Differences between saline and remifentanil ratings of intensity and unpleasantness of unmodified breathing and breathlessness were tested using a one-tailed, paired *t*-test, based on the extensive literature surrounding the analgesic actions of acutely administered opioids. Differences in intensity and unpleasantness scores were also correlated with differences in sedation, tension and discontentment between saline and remifentanil with Bonferroni corrections for multiple comparisons. Questionnaires were scored according to their respective manuals.

#### Physiological data

Physiological recordings were exported to MatLab (MathWorks Inc., Natick, MA, USA) and analysed using custom-written scripts.

### FMRI analysis

#### Image pre-processing

FMRI data processing was carried out using FEAT (FMRI Expert Analysis Tool) Version 6.00, part of FSL (FMRIB's Software Library, www.fmrib.ox.ac.uk/fsl), using a whole-brain approach. Pre-processing of the data was performed with MCFLIRT motion correction (Jenkinson et al., 2002), non-brain removal using BET (Smith, 2002), spatial smoothing using a full-width-half-maximum Gaussian kernel of 5 mm, and high pass temporal filtering (Gaussian-weighted least-squares straight line fitting, with sigma=75.0 s) with 150 s cut-off. Registration to the high resolution structural was carried out using boundary-based-registration (BBR; FSL software library), registration from high resolution structural to standard space was then further refined using nonlinear registration (FNIRT; FSL software library).

The FMRI scans were corrected for motion, scanner and cerebro-spinal fluid artefacts using independent component analysis (ICA) denoising (Kelly et al., 2010). BOLD images were corrected for physiological noise with physiological noise modelling integrated in FSL (RETROICOR) (Brooks et al., 2008, Harvey et al., 2008). The noise signal determined by RETROICOR was adjusted for interactions with the ICA denoising to ensure artefactual signal was not reintroduced through the combination of both noise correction techniques (Faull et al., 2016). This is described in detail below.

### Further details of physiological noise correction

To ensure noise was not reintroduced through the combination of ICA denoising and RETROICOR, the following stepwise process was followed:

1. ICA was used to decompose FMRI data into different spatial and temporal components using FSL's MELODIC (Multivariate Exploratory Linear Optimised Decomposition into Independent Components) using automatic dimensionality estimation. This is a data-driven Bayesian approach to ICA dimensionality estimation that allows MELODIC to choose the number of components into which the FMRI data is decomposed. This means that different FMRI scans may be decomposed into a different number of components. The noise components (classified as movement, cerebral spinal fluid (CSF) and scanner artefact) were then identified based on spatial location of signal and signal frequency (Griffanti et al., 2014, Salimi-Khorshidi et al., 2014), and manually removed from the 4D FMRI data. This is referred to as the "ICA denoised data".
2. The pulse oximetry and respiratory flow measurements allowed determination of cardiac and respiratory phase with each acquired slice of each FMRI image volume. This assigned phase is then entered into a low-order Fourier expansion (Brooks et al., 2013, Harvey et al., 2008) to derive time course regressors (2 cardiac, 2 respiratory and 1 interaction regressor). These regressors explain potential signal changes associated with cardiac and respiratory (or interacting) processes. These regressors were regressed against the ICA denoised data using FSL's Physiological Noise Modelling (PNM) tool (Brooks et al., 2008, Harvey et al., 2008). The residuals from this regression were subtracted from the ICA denoised data. This is referred to as the "PNM signal".
3. To combine the ICA denoising with PNM it is necessary to remove any overlap between the two. Therefore the PNM signal was also run through ICA denoising (Griffanti et al., 2014, Salimi-Khorshidi et al., 2014) and the resulting denoised PNM signal was removed from the ICA denoised data to give the final denoised data used in the analysis.

#### Statistical analysis

An overview of the statistical analysis is depicted in Supplementary Fig. 2.

#### First level analysis

Linear models were used to describe the data. The first level analysis used a general linear modelling (GLM) approach with multiple explanatory variables (EVs). Individual subject contrasts were generated for mild and strong anticipation periods from the beginning of the symbol presentation until the onset of the inspiratory resistive loading. ‘On’ loading periods for each of the mild and strong stimuli were then constructed from the onset of the resistance stimulus until the end of the loading period. Unloaded periods were modelled for the duration of the unloaded stimulus. Periods when participants rated their preceding sensations and the 5-s periods after each respiratory manipulation (fixation cross; presented to give participants a chance to recover before giving their subjective ratings) were modelled as regressors of no interest. P_ET_CO_2_ was entered as a separate EV in order to account for residual fluctuations in CO_2_ that could affect BOLD signal (Wise et al., 2007).

To account for possible changes in the haemodynamic response function (HRF), including slice-timing delays, external timing files were convolved with an optimal basis set of three waveforms (FLOBS: FMRIB's Linear Optimal Basis Sets, default FLOBS supplied in FSL (Woolrich et al., 2004a)), instead of the standard gamma waveform. The second and third FLOBS waveforms, which model the temporal and dispersion derivatives, were orthogonalised to the first waveform, of which the parameter estimate was then passed up to the higher level to be used in the group analysis.

#### Saline analysis

Firstly, a higher-level mixed effects analysis was conducted to obtain group average results during the saline condition only. FMRIB's Local Analysis of Mixed Effects (FLAME 1+2 (Woolrich et al., 2004b)) was used to process images with automatic outlier de-weighting, and a univariate analysis of the group mean was performed. Analysis was corrected for multiple comparisons across the whole brain. A cluster threshold of >2.3 and a corrected cluster significance threshold of p=0.05 were used.

#### Remifentanil-saline difference analysis

The difference between saline and remifentanil conditions was calculated using a two-step higher level approach. In a second-level analysis, the effects of remifentanil and saline condition were determined for the EVs relating to each of the mild and strong anticipation and loading conditions for each participant using a fixed effects analysis. This enabled contrasts between the different EVs to be created (see Supplementary Fig. 2). The resulting contrasts were fed into a third-level mixed effects analysis in order to obtain group averages. FMRIB's Local Analysis of Mixed Effects (FLAME 1+2 (Woolrich et al., 2004b)) was used to process images with automatic outlier de-weighting. EVs of no interest were the sedation score from the Bond-Lader mood scale, order of drug administration (i.e. whether participants received remifentanil or saline during their first FMRI session), and the mean difference in unpleasantness score between remifentanil and saline for each subject. CBF values obtained from the ASL (or difference in case of the saline remifentanil contrast) were included as a voxel-wise regressor of no-interest to account for remifentanil-induced perfusion changes (Pattinson et al., 2009). Analysis was corrected for multiple comparisons across the whole brain. A cluster threshold of >2.3 and a corrected cluster significance threshold of p=0.05 were used.

To test our hypothesis that opioid action in the amygdala and hippocampus modulates associative learning, a small volume corrected non-parametric region-of-interest (ROI) analysis was conducted using FSL's Randomize tool (Winkler et al., 2014), cluster corrected with Z>2.3 and p<0.05. Analysis was confined to a single bilateral mask of the hippocampus and amygdala (Harvard Oxford Subcortical Atlas thresholded at 50%), to investigate whether the anticipation of strong loading (> breathlessness) correlates with remifentanil-induced changes in unpleasantness scores.

#### Mild loading condition

The mild stimulus was inconsistently rated; some subjects found it aversive and became conditioned to the stimulus, whereas for others it was imperceptible. As we were interested in consistently conditioned stimuli for this study, data from the anticipation of mild loading is not reported. Therefore the discussion focuses on the results of the experimental breathlessness induced during the strong loading condition.

### ASL analysis

#### Generation of blood-perfusion maps

Blood flow velocities for the left and right carotid arteries were extracted from the phase contrast scan with a custom-written MatLab script. Both arteries showed a 20% perfusion increase with remifentanil compared to saline (from 31 l/s to 36 l/s, p=0.005). This was used as a correction factor in the ASL analysis (Aslan et al., 2010).

Multi-TI ASL data was corrected for physiological noise using RETROICOR (Glover et al., 2000) with a custom-written script that corrected multi-TI data by separating the time points by TI and by tag and control condition, before combining them back together. A quantitative perfusion map was generated for each session with a custom-written MatLab script that ran registration with FLIRT (FSL software library) and estimation of CBF with BASIL (Bayesian Inference for Arterial Spin Labeling MRI; a toolbox of FSL) (Okell et al., 2013). The resulting voxel-wise CBF averages for each session were incorporated into higher-level FMRI analyses to help account for any confounding effects of global CBF changes with remifentanil on the BOLD signal (Pattinson et al., 2009).

## Results

### Behavioural and physiological data

Table 1 illustrates respiratory parameters and pulse rate. Importantly, this demonstrates successful conditioning to the breathlessness (strong loading) condition by virtue of a small but significantly increased mouth pressure amplitude during anticipation of the strong stimulus compared with baseline. This represents increased respiratory effort. As expected, remifentanil induced mild respiratory depression (raised baseline P_ET_CO_2_ and decreased respiratory mouth pressure).

Blood pressure was unchanged between saline (systolic 124(±13) mmHg, diastolic 68(±8) mmHg) and remifentanil (systolic 126(±13) mmHg, diastolic 68(±10) mmHg), p=0.79 (systolic), p=0.91 (diastolic)). Resting heart rate (obtained from blood pressure cuff between scans) was unchanged: saline 67(±11) min^−1^, remifentanil 65(±7) min^−1^, p=0.35. Oxygen saturations were as follows: saline 98(±1), remifentanil 99(±1), p=0.13. Importantly, no episodes of hypoxaemia were noted at any point in the study. Continuous heart rate data (from the pulse oximeter) was unavailable during scanning on 4 occasions (2 remifentanil, 2 saline, in different subjects) therefore the heart rate data presented in Table 1 is from the 15 subjects with complete data sets.

**Fig. 2:**
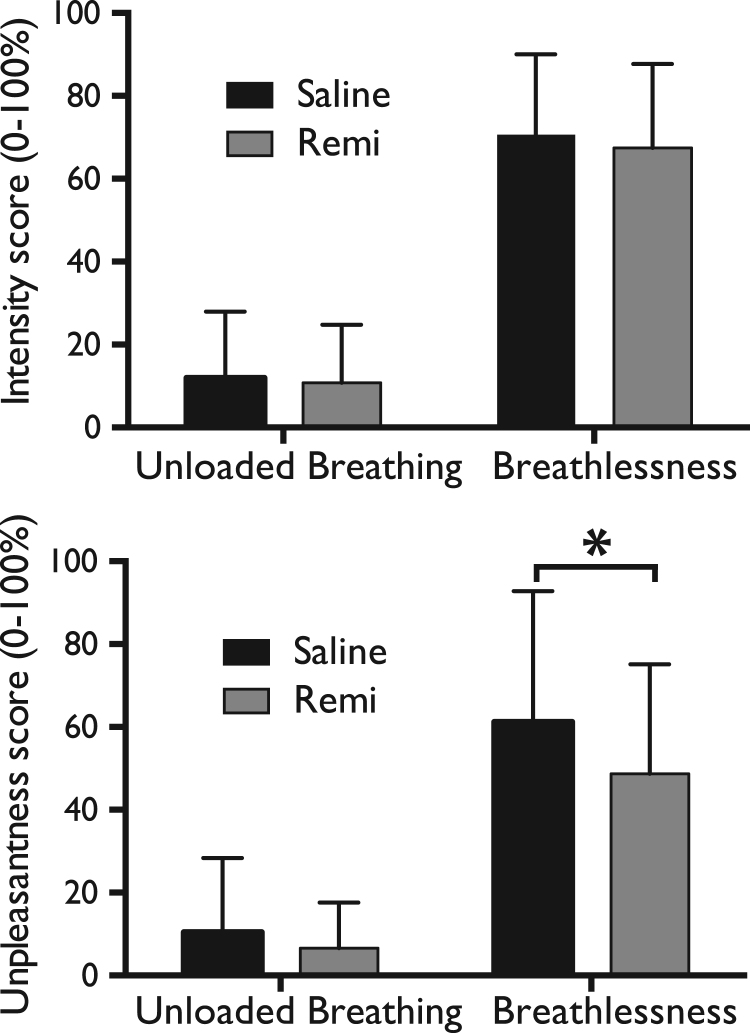
Breathlessness intensity and unpleasantness scores (on a visual-analogue scale of 0–100%) during unloaded breathing and breathlessness during both saline and remifentanil (Remi) administration. Bars represent group mean, and error bars standard deviation. *Significantly different between the groups (p<.05).

Subjective ratings of perceived intensity and unpleasantness of respiration across stimuli confirmed the successful induction of breathlessness (Table 1). Remifentanil significantly reduced unpleasantness scores during strong loading (saline vs. remifentanil: 61.2 (±31.6) vs. 48.7 (±26.2); p=0.03), but not during unloaded breathing (saline vs. remifentanil: 10.4 (±17.9) vs. 6.6 (±11.0); p=0.13) (Fig. 2). However, remifentanil did not significantly change breathlessness intensity during unloaded breathing (saline vs. remifentanil (mean VAS%: 11.9 (±16.1) vs. 10.8 (±14.0); p=0.22) or strong loading (saline vs. remifentanil: 70.5 (±19.5) vs. 67.5 (±20.2); p=0.21).

The Bond-Lader mood scales demonstrated greater sedation during remifentanil infusion (interaction effect, df=1, F=19.799, p<0.0001). There was also an interaction effect showing reduced tension (df=1, F=15.732, p=0.001) and reduced discontentment (df=1, F=7.748, p=0.012; all Bonferroni corrected, significant when p<0.0125) during the remifentanil condition (see Supplementary Fig. 3). The difference in perceived intensities between the saline and remifentanil conditions positively correlated with perceived unpleasantness (r=0.589, p=0.008), but did not correlate with changes in sedation (r=−0.086, p=0.73), tension (r=0.511, p=0.026) or discontentment (r=0.319, p=0.18), all Bonferroni corrected (significant when p<0.0125; see Supplementary Fig. 3).

#### Baseline psychological questionnaires

All participants scored within the normal range for trait anxiety (STAI-T mean: 7, SD ± 6, range (23–57)) and depression (CES-D mean: 34, SD ± 9, range (0–23)). Thus, no participants were excluded due to undiagnosed anxiety or depressive disorder.

### FMRI results

#### Conditioned anticipatory response

##### Saline

**Fig. 3:**
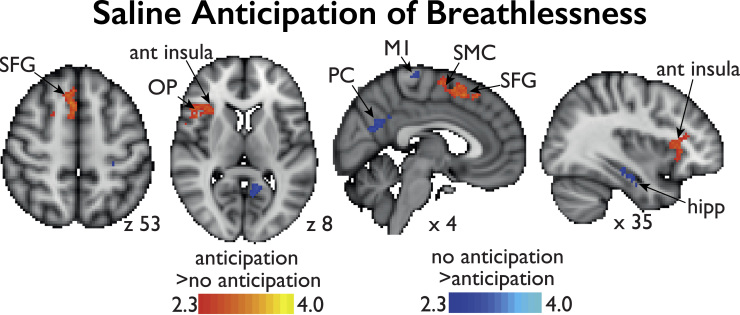
BOLD activation contrasting anticipation of breathlessness with anticipation of no loading during saline administration. The image consists of a color-rendered statistical map superimposed on a standard (MNI) brain. Significant regions are displayed with a threshold of Z>2.3 with a cluster probability threshold of p<0.05 (corrected for multiple comparisons). Abbreviations: ant insula, anterior insula; OP, opercular-frontal cortex (operculum); SMC, supplementary motor cortex; SFG, superior frontal gyrus; PC, precuneus; hipp, hippocampus; M1, primary motor cortex. No significant reductions in anticipation of loading were observed with the administration of remifentanil (not shown).

Anticipation of the strong resistive inspiratory load (contrast: strong cue – unloaded cue) increased BOLD signal in the right anterior insula and operculum, supplementary motor cortex and superior frontal gyrus, and left cerebellum (Crus I, II, VI, VII and vermis). Deactivations were observed in the right hippocampus and temporal gyrus, left precuneus, posterior cingulate cortex and bilateral primary motor and sensory cortices (Fig. 3).

##### Saline vs remifentanil

During anticipation (contrast: strong cue - unloaded cue), there were no significant mean changes in BOLD activity as a result of remifentanil administration.

##### Remifentanil-induced changes in unpleasantness

**Fig. 4:**
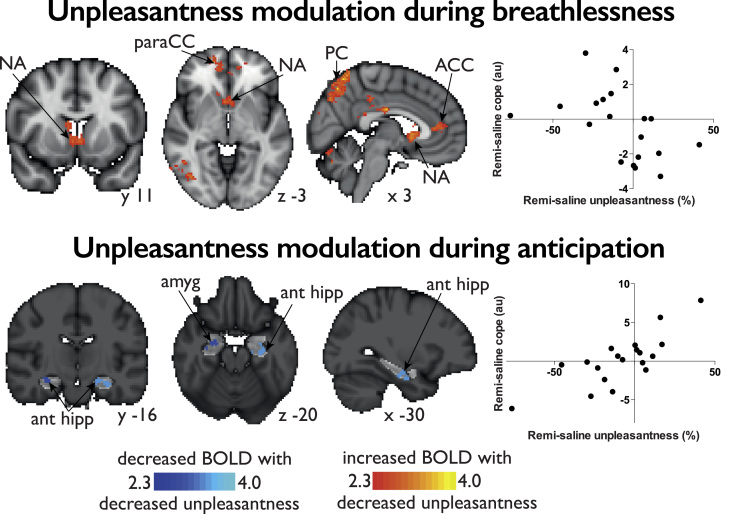
BOLD activity corresponding with remifentanil-induced decreases in unpleasantness scores across subjects. Top: Increases in BOLD activity with remifentanil that negatively correlate with decreases in breathlessness unpleasantness, with a graphical depiction of the contrast of parameter estimate (COPE) from the significant activity in the rostral ACC plotted against the decrease in unpleasantness scores. Bottom: Decreases in BOLD activity with remifentanil that correlate with decreases in unpleasantness during anticipation of strong loading in the hippocampus and amygdala, with a graphical depiction of the contrast of parameter estimate (COPE) from the combined significant activity plotted against the decrease in unpleasantness scores. The image consists of a color-rendered statistical map superimposed on a standard (MNI) brain. The bright gray region delineates the region of interest analysed. Abbreviations: ACC, anterior cingulate cortex; NA, nucleus accumbens; paraCC, paracingulate cortex; prefrontal cortex; PC, precuneus; amyg, amygdala; ant hipp, anterior hippocampus.

Activity within the bilateral anterior hippocampus and right amygdala correlated with the remifentanil-induced changes in unpleasantness scores across subjects during anticipation (contrast: strong cue – unloaded cue), within the bilateral ROI of the amygdala and hippocampus (Fig. 4).

#### Breathlessness

##### Saline

**Fig. 5:**
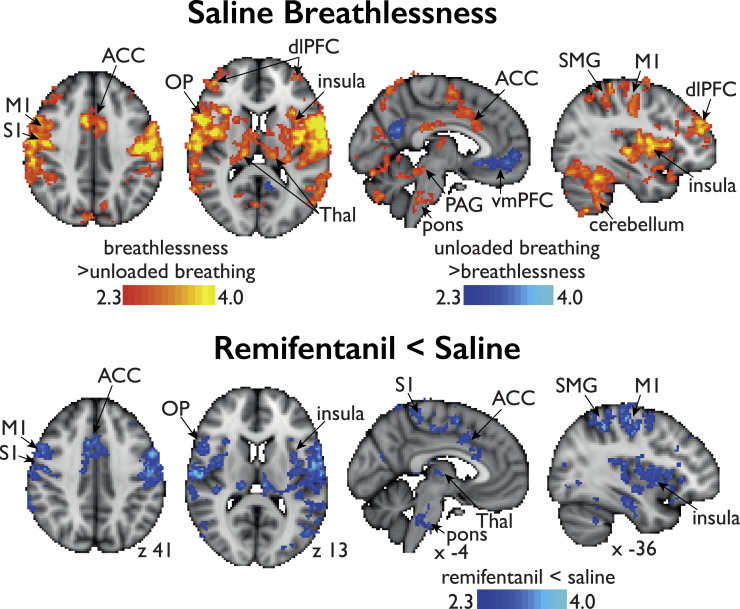
BOLD activity during the breathlessness condition during saline (placebo) infusion (top figure) and the effect of remifentanil infusion (lower figure). The image consists of a color-rendered statistical map superimposed on a standard (MNI) brain. Significant regions are displayed with a threshold of Z>2.3 and a cluster probability threshold of p<0.05 (corrected for multiple comparisons). Abbreviations: dlPFC, dorsolateral prefrontal cortex; OFC, opercular-frontal cortex (operculum); ACC, anterior cingulate cortex; M1/S1 primary motor and sensory cortices; SMG, supramarginal gyrus; Thal, thalamus.

Mean increases in BOLD activity during breathlessness was observed in the bilateral dorsolateral prefrontal cortex, insula, operculum, anterior cingulate cortex, supplementary motor cortex, primary motor and sensory cortices, supramarginal gyrus, superior parietal lobule, amygdala, thalamus, caudate nucleus, caudal portion of the periaqueductal gray, pons and cerebellum (Crus I, VI, VII, VIII) (Fig. 5). Mean decreases in BOLD activity were observed in the ventromedial prefrontal cortex and precuneus.

##### Saline vs. remifentanil

Mean decreases in BOLD activity as a result of remifentanil administration were observed within the cortical network associated with breathlessness during the saline condition. These areas included bilateral insula, operculum, anterior cingulate cortex, supplementary motor cortex, primary motor and sensory cortices, supramarginal gyrus, thalamus, pons and cerebellum (left Crus I and VI) (Fig. 5). No increases in BOLD activity were observed during breathlessness as a result of remifentanil administration compared to the saline condition.

##### Remifentanil-induced changes in unpleasantness

A network of areas that were anti-correlated with the remifentanil-induced decreases in breathlessness unpleasantness were identified, i.e. remifentanil-induced decreases in breathlessness unpleasantness were matched with corresponding increases in BOLD activity (Fig. 4). These areas included the rostral anterior cingulate cortex, nucleus accumbens, ventromedial prefrontal cortex, precuneus, posterior cingulate cortex, supramarginal gyrus and cerebellum (right VI).

#### Arterial spin labeling

Remifentanil increased global grey matter cerebral blood flow by approximately 10% compared to saline (saline grey matter CBF=40.7 (4.4) ml/100 g/min, remifentanil grey matter CBF= 44.8 (4.2) ml/100 g/min, p=0.003).

## Discussion

### Key findings

Remifentanil significantly reduced the unpleasantness but not the intensity of breathlessness, indicating the ability of low-dose opioids to dissociate breathlessness intensity from unpleasantness.

Conditioned anticipation of breathlessness was associated with brain activity in areas including the right anterior insula and operculum, supplementary motor cortex and superior frontal gyrus. In the amygdala and hippocampus, remifentanil suppressed anticipation-evoked neural activity, which correlated positively with reduction of breathlessness unpleasantness.

Increased activity was observed during breathlessness in several brain areas including the bilateral dorsolateral prefrontal cortex, insula, anterior cingulate cortex, amygdala, thalamus, pons and areas of the periaqueductal gray. Remifentanil suppressed activity within the neural network associated with breathlessness, including the insula, anterior cingulate cortex, thalamus, primary sensory and motor cortices and pons.

Furthermore, during breathlessness remifentanil induced decreases in unpleasantness correlated with increases in activity in areas such as the rostral anterior cingulate cortex (rACC) and nucleus accumbens.

### Anticipation of breathlessness

It is recognized that anticipation triggers a set of neural events that primes and influences the response to the upcoming aversive stimuli. In our study, anticipation of breathlessness increased the activity in the right insula and the adjacent operculum. The anterior insula is functionally associated with emotional processing and self-awareness (Craig, 2009) and is one of the most commonly activated regions during anticipation of aversive stimuli. It is thought to facilitate the detection of saliency and mediate the anticipatory effects on aversive stimuli (Atlas et al., 2010, Palermo et al., 2015, Ploner et al., 2010). Based on the present data, we speculate that the anterior insula fulfills the same role in breathlessness.

### Opioid effects on anticipation

We observed an opioid-induced activity reduction in the amygdala and hippocampus during the anticipation period, which correlated with a reduction of breathlessness unpleasantness. The amygdala is a key player in emotional processing of aversive stimuli and is implicated in conditioned aversive learning (Phelps and LeDoux, 2005). Its interaction with the hippocampus, a structure pivotal for memory consolidation (Eichenbaum and Lipton, 2008), has been shown to enhance the encoding and consolidation of the learned association between neutral (anticipatory cues) and aversive stimuli in the hippocampus (LaBar et al., 1995, Phelps, 2004). Furthermore, these areas are rich in endogenous opioids and play a role in many other opioid-related tasks including reward processing (Sesack and Grace, 2010) and opioid mediated analgesia (Favaroni Mendes and Menescal-de-Oliveira, 2008, McGaraughty and Heinricher, 2002). The endogenous opioid system plays a key role in forming associations between neutral stimuli and aversive stimuli; opioid agonists attenuate and antagonists facilitate the acquisition of such associations (Eippert et al., 2008, Fanselow, 1998, McNally et al., 2004). We found that opioid suppression of breathlessness unpleasantness is related to the opioid induced suppression of activity in the amygdala and hippocampus during the preceding anticipatory cue. Therefore, it is conceivable that opioid relief of chronic breathlessness stems (at least in part) from functional interference of neural activity in brain areas that regulate emotional and memory functions related to breathlessness anticipation.

### Brain activations during breathlessness

We identified a network of areas activated during breathlessness (Fig. 5) in line with previous published work (Herigstad et al., 2011, Pattinson and Johnson, 2014). This includes the dorsolateral prefrontal cortex, anterior cingulate cortex, insula, amygdala and the caudate, areas that are known to represent cognitive and emotional responses to aversive stimuli (Wiech et al., 2008, Wiech and Tracey, 2009), as well the motor and sensory cortices most likely representing the motor response and sensory intensity of breathlessness (Hayen et al., 2012). These findings further support the growing concept that perception of breathlessness is a multi-dimensional sensory experience (De Peuter et al., 2004, Janssens et al., 2009) encompassing sensory, cognitive and affective dimensions. Remifentanil suppressed activity in selected regions representing all the three domains. Remifentanil suppressed breathlessness-induced activity in the pons, an area known to contain nuclei of respiratory control (Pattinson, 2008), and further work using brainstem focused sequences would be merited to understand this in more depth (Faull et al., 2015, Faull et al., 2016).

Remifentanil-induced suppression of unpleasantness of breathlessness was associated with increased activity in the rACC, and nucleus accumbens. The rACC is part of the endogenous opioid system (Jones et al., 1991) with a well-documented role in modulating the perception of aversive stimuli in response to exogenous opioids as well as in response to cognitive manipulations (Eippert et al., 2009, Petrovic et al., 2002). PET imaging has shown that increased opioidergic activity in rACC and the nucleus accumbens reduced the affective and the sensory perception of a sustained aversive stimulus (Zubieta et al., 2001). Therefore, it is likely that opioids reduced the unpleasantness of the breathlessness by engaging the rACC and nucleus accumbens, structures of the endogenous opioid system that have been shown to modulate the unpleasantness of other aversive stimuli, such as pain.

### Opioid effects on physiological measures of respiration

Infusion of remifentanil was associated with mild respiratory depression; a well-known effect of opioids (Mitsis et al., 2009, Pattinson, 2008, Pattinson et al., 2007). However, we observed a significantly increased mouth pressure amplitude during anticipation of breathlessness when compared with anticipation of no load for both saline and remifentanil conditions. This suggests that the conditioned increase in ventilation in response to the anticipatory cue was maintained during both conditions. This behavioural finding is in accordance with maintained mean BOLD activity during anticipation. Based on our data showing suppression of activity in emotion processing areas, we speculate that low dose opioids dissociate respiratory and emotional effects whilst maintaining the conditioned ventilatory anticipatory response.

### Opioid effects upon emotional state

During remifentanil infusion, participants were in a more positive emotional state, reporting reduced tension and discontentment and increased sedation. Opioids cause a generalized positive shift in affect across the hedonic spectrum, from enhancing the pleasantness of food to decreasing perceived pain (Leknes and Tracey, 2008). By accounting for these factors in our FMRI analysis, we are confident that the observed BOLD changes were specific to breathlessness and its anticipation.

### Clinical relevance

Our findings demonstrate how studies performed in healthy volunteers help probe mechanisms that might otherwise be difficult to isolate in clinical populations. A recent FMRI study in COPD (Herigstad et al., 2016, Herigstad et al., 2015) demonstrated brain activations in the insula, prefrontal cortices, operculum and striatum in response to breathlessness-related word cues. The authors speculated that these activations might relate to a learned or conditioned response. The present study adds weight to that speculation by demonstrating similarities in the anticipatory brain activation pattern (during saline infusion) in an experiment in which conditioned learning was specifically induced to model aspects of chronic breathlessness.

There is an unmet clinical need for new treatments for chronic breathlessness (Currow et al., 2016). Targeting associative learning represents an untapped potential for new treatments. The present data demonstrates that conditioned anticipatory activity is mediated by the anterior insula, one of the key brain structures associated with emotion regulation (Craig 2009). In health, anticipatory brain activity is a beneficial adaptation to avoid danger and suffering (Ley, 2001), but if overexpressed may lead to unhealthy deconditioning, activity avoidance, worsening quality of life and worsening breathlessness (Hayen et al., 2013). Opioid effects on associative learning might explain how low-dose opioids may have gradual increases in efficacy over the first week of administration (Currow et al., 2013, Oxberry et al., 2013). Opioid side effects are well described (Pattinson, 2015, Ray et al., 2016, Robertson et al., 2016, Vozoris et al., 2016), with death due to respiratory depression particularly feared. An in-depth understanding of their mechanisms of action will pave the way to safer alternatives. Additionally, maladaptive learning is starting to be recognized as an important factor in chronic pain, with generalization of negative emotions over sensory discrimination contributing to chronicity (Zaman et al., 2015). Health anxiety often co-exists with disease, exacerbating symptoms and morbidity. The importance of this has only recently been realized in mainstream medical journals (Tyrer et al., 2016). It is likely that such mechanisms play a similarly important role in chronic breathlessness, where they could mediate the disease trajectory by initiating and fuelling fear, activity avoidance and deconditioning.

### Discussion of methods

Respiratory manipulations and opioid infusions may confound the interpretation of BOLD FMRI (Pattinson and Wise, 2016). Therefore, we have included a number of steps in the study design and analysis to address these potential confounds. These are discussed below and in more detail in the Supplementary material.

#### Control of end-tidal gases

Inspiratory resistive loading may alter PetCO_2_ (Hayen et al., 2015), which confounds interpretation of BOLD responses (Cohen et al., 2002, Wise et al., 2007). Respiratory depression caused by remifentanil may also lower oxygen saturations, again potentially confounding BOLD. Therefore, we manually controlled both PetCO_2_ and PetO_2_ on a breath-by-breath basis to minimise stimulus-correlated fluctuations. To do this, it was necessary to perform the experiment upon a baseline of mild hypercapnia (+0.3 kPa above baseline), and mild hyperoxia (PetO_2_ of 20 kPa). The caveat with this approach is that this mild hypercapnia and hyperoxia will slightly reduce BOLD responsiveness overall, and is thus conservative.

#### The use of ASL to account for between-session differences in CBF

Remifentanil depresses respiration (Mitsis et al., 2009), leading to hypercapnia and increased baseline CBF (MacIntosh et al., 2008). Voxel-wise measures of CBF were incorporated as a covariate, allowing for differing effects of baseline CBF on BOLD response across the brain (Pattinson et al., 2009), thus accounting for additive or subtractive differences in stimulus-evoked BOLD response. An important caveat of this technique is that it assumes a linear effect of CBF on baseline BOLD and a more complex relationship between CBF and stimulus-evoked BOLD might not be accounted for.

#### Accounting for potential changes in the haemodynamic response

The haemodynamic response function (HRF) varies between brain regions and individuals (Handwerker et al., 2004), and may be influenced by varying levels of neurotransmitters (Muthukumaraswamy et al., 2012), and by exogenously administered drugs (Luchtmann et al., 2010). Therefore, instead of using the fixed standard gamma waveform, we used an optimal basis set of three waveforms (FLOBS: FMRIB's Linear Optimal Basis Sets, default FLOBS supplied in FSL (Woolrich et al., 2004a)). Although this approach may more accurately account for varying HRFs, it may lead to a small underestimation of the effect size.

#### Physiological noise correction

The main sources of physiological noise in FMRI relate to the respiratory and cardiac cycle (Brooks et al., 2013, Glover et al., 2000, Harvey et al., 2008). These were corrected using RETROICOR and ICA-based noise classification. Whilst cleaning imaging data of unwanted noise, the main caveat for noise correction in general is that any neural signals that are aliased to the cardiac and respiratory cycles will be lost. RETROICOR uses continuous measures of cardiac and respiratory cycles during a scan to regress out these known quantities. It requires hardware investment and technical expertise at the time of scanning. Lost signal (e.g. a poor pulse oximeter trace) cannot be retrospectively accounted for. ICA decomposition requires training data which has to be classified manually, which is time consuming and can be subject to experimenter bias. In the present study, the person who did the ICA classification was blinded to experimental condition. The benefit of using a combination of RETROICOR and ICA decomposition is a comprehensive noise correction, but this has the caveat of requiring an even more complex analysis to ensure that noise is not reintroduced.

## Conclusions

Anticipation of cues associated with breathlessness activates the right anterior insula and the adjacent operculum, brain regions implicated in aversive conditioning and emotional regulation. Remifentanil suppressed the neural activity during the anticipatory period in the amygdala and hippocampus, regions implicated in aversive memory formation, which was expressed as reductions in unpleasantness of breathlessness. Furthermore, during remifentanil infusion, reductions in breathlessness unpleasantness correlated with increases in activity in the rACC and nucleus accumbens, well-established components of the endogenous opioid system known to decrease the perception of aversive stimuli. These findings suggest that opioids palliate breathlessness through an interplay of altered associative learning mechanisms, independent of or in addition to effects on brainstem respiratory control. These provide potential targets for novel or improved behavioural or pharmacological treatments to break the well-known spiral of decline in order to ease daily suffering of people with chronic breathlessness.

## Funding/support

The study was funded by the Medical Research Council (United Kingdom) as part of a Clinician Scientist Fellowship awarded to Dr Pattinson [G0802826]. AH, PG, VW and KP were supported by the National Institute for Health Research Oxford Biomedical Research Centre based at Oxford University Hospitals NHS Trust and University of Oxford.

## Conflict of interest

KP has acted as a consultant for Nektar Therapeutics.

## Figures and Tables

**Table 1:** Effects of resistive load breathlessness on respiratory parameters. P_ET_CO_2_=partial pressure of end-tidal carbon dioxide. P_ET_O_2_=partial pressure of end-tidal oxygen.

	**Saline**				**Remifentanil**			
**Variable**	**Anticipation unloaded**	**Unloaded breathing**	**Anticipation breathlessness**	**Strong loading**	**Anticipation unloaded**	**Unloaded breathing**	**Anticipation breathlessness**	**Strong loading**
Mouth pressure amplitude [cmH_2_O]	2.7 (0.7)	2.4 (0.5)	3.5 (1.7)¶	12.7 (4.1)*	2.2 (0.5)	2.0 (0.4)	2.8 (1.0)∆	10.9 (3.4)*
P_ET_CO_2_ [kPa]	5.5 (0.6)	5.6 (0.6)	5.5 (0.5)	5.5 (0.6)	6.0 (0.6)*	6.1 (0.6)*	6.0 (0.6)*	6.0 (0.6)*
P_ET_O_2_ [kPa]	20.0 (0.9)	19.8 (0.8)	19.9 (0.7)	20.2 (0.8)	20.1 (1.1)	19.8 (1.0)	19.9 (0.9)	20.3 (1.2)
Intensity rating [%VAS]		12(16)		71(20)*		11(14)		68(20)§*
Unpleasantness rating [%VAS]		10(18)		61(32)*		7(11)		49(26)*§†
Heart rate [min^-1^] (N=15)	68 (11)	67 (10)	68 (11)	69 (11)	68 (11)	65 (10)	69 (11)	66 (9)

Values are presented as mean (SD). N=19.

Complete heart rate data in each epoch only available for 15 subjects.

* Significantly different from saline unloaded breathing at p<0.001.

§ Significantly different from remifentanil unloaded breathing at p<0.001.

† Significantly different from saline breathlessness at p<0.05.

¶ Significantly different from saline anticipation unloaded breathing at p<0.05.

∆ Significantly different from remifentanil anticipation unloaded breathing at p<0.05.
