# Supplementary material for: Opioid suppression of conditioned anticipatory brain responses to breathlessness
Source: Neuroimage. 2017 Apr 15;150:383–94. doi: 10.1016/j.neuroimage.2017.01.005 (PMC5391989; doi:10.1016/j.neuroimage.2017.01.005)
Supplement: Supplementary file 1 — Supplementary material [file mmc1.docx]

**Opioid suppression of anticipatory brain responses to breathlessness**

Anja Hayen, Vishvarani Wanigasekera, Olivia K Faull, Stewart F Campbell, Payashi S Garry, Simon J.M. Raby, Josephine Robertson, Ruth Webster, Richard G Wise, Mari Herigstad, Kyle T. S. Pattinson

**Online data supplement**

**

Supplementary Figure 1. Schematic diagram of respiratory circuit. A facemask (Hans Rudolph, Kansas City, MO, USA) connects to a bacterial and viral filter (Vitalograph, Buckingham, UK) from which respiratory gases and respiratory pressure are sampled via polyethylene extension tubing (Vygon SA, Ecouen, France). One sampling line leads to a pressure transducer (MP 45, ± 50 cmH_2_O, Validyne Corp., Northridge, CA, USA) connected to an amplifier (Pressure transducer indicator, PK Morgan Ltd, Kent, UK). The second sampling line connects to a gas analyzer that samples O_2_ and CO_2_ (ADInstrument Ltd, Oxford, UK). A one-way valve allows expired air to escape close to the mouth in order to minimize rebreathing (Hans Rudolf, Kansas City, MO, USA). The breathing system contains three arms. Participants usually breathe through the first, unobstructed arm. This arm can be closed off by inflating a balloon (embolectomy balloon, Microtek Medical B.V., Zutphen, Netherlands) via a hydraulic system. Closure of the arm forces participants to breathe through the second arm, which contains a pediatric respiratory filter (mild resistor, Intersurgical, Wokingham, UK) and a second balloon valve. Closure of both valves forces breathing through a third arm, which contains a porous glass disk (strong resistor). The three arms recombine into a spirometry module that records respiratory flow (ADInstruments Ltd, Oxford, UK) connected to a custom-made mixing chamber in which medical air, oxygen and 10% CO_2_ in air are combined.

**

Supplementary Figure 2. Overview of statistical analysis performed within FEAT for Remifentanil > Saline. Abbreviations: R-S, remifentanil - saline; UNP, unpleasantness; ASL, arterial spin labeling; P_ET_CO_2_, pressure of end-tidal carbon dioxide.

**SUPPLEMENTARY RESULTS**

*Perception of the mild respiratory load:* During the saline session, the mild stimulus received an average intensity rating of 31.5 (20.7) %VAS and an average unpleasantness rating of 24.8 (25.0) %VAS. During the remifentanil session, the mild stimulus received an average intensity rating of 23.4 (16.3) %VAS and an average unpleasantness rating of 10.8 (13.3) %VAS.

**

Supplementary Figure 3. Sedation, tension and contentment (3 dimensions of the Bond-Lader questionnaire) 15 minutes before the infusion (pre-infusion), and during the infusion (infusion) for saline and remifentanil. Data are mean ± standard deviation for 19 participants. ** p<.01, *** p<.001. Abbreviation: VAS, visual analogue scale.

**SUPPLEMENTARY DISCUSSION**

*Discussion of methods.*

Respiratory manipulations and opioid infusions may confound the interpretation of BOLD FMRI. Therefore we have included a number of steps in the study design and analysis to address these potential confounds.

*Control of end-tidal carbon dioxide (PetCO_2_).* As we have studied healthy young adults without lung disease (and thus normal diffusion of CO_2_ across the lung alveolus), it is standard to assume that in healthy lungs changes in end-tidal CO_2_ (PetCO_2_) reflect changes in arterial CO_2_ (PaCO_2_). A decreased PaCO_2_ will enhance the stimulus-evoked BOLD response, whereas an increased PaCO_2_ will dampen evoked BOLD responses (Cohen et al., 2002). Inspiratory resistive loading may either increase or decrease end-tidal CO_2_ depending on the individual response (Hayen et al., 2015), leading to an unpredictable stimulus-correlated effect on PaCO_2_, thus introducing noise and potential bias to the statistical analysis. Controlling PetCO_2_ with end-tidal forcing as in the present minimising such stimulus-correlated confounds (Wise et al., 2007), residual fluctuations in PetCO_2_ were modelled in the first-level FMRI analysis. In order to be able to adjust PetCO_2_ both upwards and downwards, it is necessary to perform the experiment upon a baseline of mild hypercapnia (we raised it by +0.3kPa). The caveat with this approach is that this mild hypercapnia will slightly reduce BOLD responsiveness overall (Cohen et al., 2002), thus incorporating end-tidal forcing is a conservative approach in that it may makes the study slightly less sensitive. Another consideration is that end-tidal forcing, either with an automated technique (Wise et al., 2007) or performed manually, is a technique that requires some training and expertise and thus adds to resource implications of a study.

*Control of end-tidal oxygen (PetO_2_)*: Changes in respiration also induce changes in arterial oxygen (PaO_2_) influencing BOLD responses (Wise et al., 2007).  The main concern in the present study was hypoxaemia (reduced oxygen saturations) due to respiratory depression caused by remifentanil. Excessive additional supplementary oxygen could also affect CBF and BOLD responsiveness. A compromise was therefore reached where we maintained baseline PetO_2_ at 20kPa at all times. This necessitated increasing the inspired oxygen concentration from 21% (i.e. room air) by 3% to approximately 24% depending on each subject's response. By maintaining PetO_2_ slightly above normal, we were confident that changes in respiration would have less effect on arterial oxygen saturations and BOLD, as the oxyhaemoglobin dissociation curve at this point is almost flat.

*The use of ASL to account for between-session differences in CBF.* Remifentanil depresses respiration (Mitsis et al., 2009) leading to hypercapnia and increased baseline CBF (MacIntosh et al., 2008). We included voxel-wise measures of CBF (measured with ASL) as a covariate, as previously described (Pattinson et al., 2009). This analysis approach assumes a linear effect of CBF on baseline BOLD, thus accounting for additive or subtractive differences in stimulus-evoked BOLD response. Whole-brain ASL coverage allows for differing effects of baseline CBF on BOLD response across different brain regions. An important caveat of this technique is that a more complex relationship between CBF and stimulus-evoked BOLD might not be accounted for.

Increased flow velocity in the arterial supply of the brain (i.e. carotid and vertebral arteries) may adversely affect ASL labelling efficiency. Therefore, we measured flow velocity in the carotid arteries with phase contrast angiography and incorporated a correction technique described by Aslan (Aslan et al., 2010). This technique could be further refined by measuring flow in all four arteries of the neck and using vessel selective territory mapping ASL (Okell et al., 2013) to correct each voxel depending on its arterial supply. This might be useful in patients with cerebrovascular disease that may affect each arterial territory differently (Kelly et al., 2013). Such further refinement is unlikely to be critical in the present study of healthy volunteers.

The main limitations of using ASL for voxel-wise correction are that additional time for scanning is required and additional resources are required for the analysis. These should be relatively straightforward in a volunteer study such as this, but consideration to patient burden would be required for clinical studies. For the phase contrast scans, a head coil would need to cover the neck area, and as phase contrast is usually acquired in neck regions it might be affected by dental work.

*Accounting for potential changes in the haemodynamic response.* The BOLD haemodynamic response function (HRF) varies between brain regions and individuals (Handwerker et al., 2004), and may be influenced by varying levels of neurotransmitters (Muthukumaraswamy et al., 2012), and by exogenously administered drugs (Luchtmann et al., 2010). Therefore instead of using the fixed standard gamma waveform, we used an optimal basis set of three waveforms (FLOBS: FMRIB's Linear Optimal Basis Sets, default FLOBS supplied in FSL (Woolrich et al., 2004a)). Although this approach may more accurately account for varying HRFs it may lead to a small underestimation of the effect size.

*Physiological noise correction.* The main sources of physiological noise in FMRI relate to the respiratory and cardiac cycle (Brooks et al., 2013; Glover et al., 2000; Harvey et al., 2008). As our experimental protocol profoundly affected respiration, rigorously corrected for physiological noise. The main caveat for noise correction in general is that any neural signals that are aliased to the cardiac and respiratory cycles will be lost. A theoretical advantage for RETROICOR is that it regresses out a known quantity that is measured. RETROICOR requires the continuous measurement of cardiac and respiratory cycles time locked with scan acquisition, thus requires hardware investment and technical expertise at the time of scanning. Lost signal cannot be retrospectively accounted for (e.g. a poor pulse oximetry trace is quite common). ICA decomposition requires training data which has to be classified manually. In addition to being time consuming, manual classification can be subject to experimenter bias. In the present study the person who did the ICA classification was blinded to experimental condition. The benefit of using a combination RETROICOR and ICA decomposition is that more comprehensive noise correction can be performed but has the caveat of requiring even a more complex analysis to ensure that noise is not reintroduced (see methods section).

Aslan, S., Xu, F., Wang, P.L., Uh, J., Yezhuvath, U.S., van Osch, M., Lu, H., 2010. Estimation of labeling efficiency in pseudocontinuous arterial spin labeling. Magn Reson Med 63, 765-771.

Brooks, J.C., Faull, O.K., Pattinson, K.T., Jenkinson, M., 2013. Physiological noise in brainstem FMRI. Front Hum Neurosci 7, 623.

Cohen, E.R., Ugurbil, K., Kim, S.G., 2002. Effect of basal conditions on the magnitude and dynamics of the blood oxygenation level-dependent fMRI response. J Cereb Blood Flow Metab 22, 1042-1053.

Glover, G.H., Li, T.Q., Ress, D., 2000. Image-based method for retrospective correction of physiological motion effects in fMRI: RETROICOR. Magn Reson Med 44, 162-167.

Handwerker, D.A., Ollinger, J.M., D'Esposito, M., 2004. Variation of BOLD hemodynamic responses across subjects and brain regions and their effects on statistical analyses. Neuroimage 21, 1639-1651.

Harvey, A.K., Pattinson, K.T., Brooks, J.C., Mayhew, S.D., Jenkinson, M., Wise, R.G., 2008. Brainstem functional magnetic resonance imaging: disentangling signal from physiological noise. J Magn Reson Imaging 28, 1337-1344.

Hayen, A., Herigstad, M., Wiech, K., Pattinson, K.T., 2015. Subjective evaluation of experimental dyspnoea--effects of isocapnia and repeated exposure. Respir Physiol Neurobiol 208, 21-28.

Kelly, M.E., Rowland, M.J., Okell, T.W., Chappell, M.A., Corkill, R., Kerr, R.S., Westbrook, J., Jezzard, P., Pattinson, K.T., 2013. Pseudo-continuous arterial spin labelling MRI for non-invasive, whole-brain, serial quantification of cerebral blood flow following aneurysmal subarachnoid haemorrhage. Transl Stroke Res 4, 710-718.

Luchtmann, M., Jachau, K., Tempelmann, C., Bernarding, J., 2010. Alcohol induced region-dependent alterations of hemodynamic response: implications for the statistical interpretation of pharmacological fMRI studies. Exp Brain Res 204, 1-10.

MacIntosh, B.J., Pattinson, K.T., Gallichan, D., Ahmad, I., Miller, K.L., Feinberg, D.A., Wise, R.G., Jezzard, P., 2008. Measuring the effects of remifentanil on cerebral blood flow and arterial arrival time using 3D GRASE MRI with pulsed arterial spin labelling. J Cereb Blood Flow Metab 28, 1514-1522.

Mitsis, G.D., Governo, R.J., Rogers, R., Pattinson, K.T., 2009. The effect of remifentanil on respiratory variability, evaluated with dynamic modeling. J Appl Physiol (1985) 106, 1038-1049.

Muthukumaraswamy, S.D., Evans, C.J., Edden, R.A., Wise, R.G., Singh, K.D., 2012. Individual variability in the shape and amplitude of the BOLD-HRF correlates with endogenous GABAergic inhibition. Hum Brain Mapp 33, 455-465.

Okell, T.W., Chappell, M.A., Kelly, M.E., Jezzard, P., 2013. Cerebral blood flow quantification using vessel-encoded arterial spin labeling. J Cereb Blood Flow Metab 33, 1716-1724.

Pattinson, K.T., Governo, R.J., MacIntosh, B.J., Russell, E.C., Corfield, D.R., Tracey, I., Wise, R.G., 2009. Opioids depress cortical centers responsible for the volitional control of respiration. J Neurosci 29, 8177-8186.

Wise, R.G., Pattinson, K.T., Bulte, D.P., Chiarelli, P.A., Mayhew, S.D., Balanos, G.M., O'Connor, D.F., Pragnell, T.R., Robbins, P.A., Tracey, I., Jezzard, P., 2007. Dynamic forcing of end-tidal carbon dioxide and oxygen applied to functional magnetic resonance imaging. J Cereb Blood Flow Metab 27, 1521-1532.

Aslan, S., Xu, F., Wang, P.L., Uh, J., Yezhuvath, U.S., van Osch, M., Lu, H., 2010. Estimation of labeling efficiency in pseudocontinuous arterial spin labeling. Magn Reson Med 63, 765-771.

Brooks, J.C., Faull, O.K., Pattinson, K.T., Jenkinson, M., 2013. Physiological noise in brainstem FMRI. Front Hum Neurosci 7, 623.

Cohen, E.R., Ugurbil, K., Kim, S.G., 2002. Effect of basal conditions on the magnitude and dynamics of the blood oxygenation level-dependent fMRI response. J Cereb Blood Flow Metab 22, 1042-1053.

Glover, G.H., Li, T.Q., Ress, D., 2000. Image-based method for retrospective correction of physiological motion effects in fMRI: RETROICOR. Magn Reson Med 44, 162-167.

Handwerker, D.A., Ollinger, J.M., D'Esposito, M., 2004. Variation of BOLD hemodynamic responses across subjects and brain regions and their effects on statistical analyses. Neuroimage 21, 1639-1651.

Harvey, A.K., Pattinson, K.T., Brooks, J.C., Mayhew, S.D., Jenkinson, M., Wise, R.G., 2008. Brainstem functional magnetic resonance imaging: disentangling signal from physiological noise. J Magn Reson Imaging 28, 1337-1344.

Hayen, A., Herigstad, M., Wiech, K., Pattinson, K.T., 2015. Subjective evaluation of experimental dyspnoea--effects of isocapnia and repeated exposure. Respir Physiol Neurobiol 208, 21-28.

Kelly, M.E., Rowland, M.J., Okell, T.W., Chappell, M.A., Corkill, R., Kerr, R.S., Westbrook, J., Jezzard, P., Pattinson, K.T., 2013. Pseudo-continuous arterial spin labelling MRI for non-invasive, whole-brain, serial quantification of cerebral blood flow following aneurysmal subarachnoid haemorrhage. Transl Stroke Res 4, 710-718.

Luchtmann, M., Jachau, K., Tempelmann, C., Bernarding, J., 2010. Alcohol induced region-dependent alterations of hemodynamic response: implications for the statistical interpretation of pharmacological fMRI studies. Exp Brain Res 204, 1-10.

MacIntosh, B.J., Pattinson, K.T., Gallichan, D., Ahmad, I., Miller, K.L., Feinberg, D.A., Wise, R.G., Jezzard, P., 2008. Measuring the effects of remifentanil on cerebral blood flow and arterial arrival time using 3D GRASE MRI with pulsed arterial spin labelling. J Cereb Blood Flow Metab 28, 1514-1522.

Mitsis, G.D., Governo, R.J., Rogers, R., Pattinson, K.T., 2009. The effect of remifentanil on respiratory variability, evaluated with dynamic modeling. J Appl Physiol (1985) 106, 1038-1049.

Muthukumaraswamy, S.D., Evans, C.J., Edden, R.A., Wise, R.G., Singh, K.D., 2012. Individual variability in the shape and amplitude of the BOLD-HRF correlates with endogenous GABAergic inhibition. Hum Brain Mapp 33, 455-465.

Okell, T.W., Chappell, M.A., Kelly, M.E., Jezzard, P., 2013. Cerebral blood flow quantification using vessel-encoded arterial spin labeling. J Cereb Blood Flow Metab 33, 1716-1724.

Pattinson, K.T., Governo, R.J., MacIntosh, B.J., Russell, E.C., Corfield, D.R., Tracey, I., Wise, R.G., 2009. Opioids depress cortical centers responsible for the volitional control of respiration. J Neurosci 29, 8177-8186.

Wise, R.G., Pattinson, K.T., Bulte, D.P., Chiarelli, P.A., Mayhew, S.D., Balanos, G.M., O'Connor, D.F., Pragnell, T.R., Robbins, P.A., Tracey, I., Jezzard, P., 2007. Dynamic forcing of end-tidal carbon dioxide and oxygen applied to functional magnetic resonance imaging. J Cereb Blood Flow Metab 27, 1521-1532.

Aslan, S., Xu, F., Wang, P.L., Uh, J., Yezhuvath, U.S., van Osch, M., Lu, H., 2010. Estimation of labeling efficiency in pseudocontinuous arterial spin labeling. Magn Reson Med 63, 765-771.

Brooks, J.C., Faull, O.K., Pattinson, K.T., Jenkinson, M., 2013. Physiological noise in brainstem FMRI. Front Hum Neurosci 7, 623.

Cohen, E.R., Ugurbil, K., Kim, S.G., 2002. Effect of basal conditions on the magnitude and dynamics of the blood oxygenation level-dependent fMRI response. J Cereb Blood Flow Metab 22, 1042-1053.

Glover, G.H., Li, T.Q., Ress, D., 2000. Image-based method for retrospective correction of physiological motion effects in fMRI: RETROICOR. Magn Reson Med 44, 162-167.

Handwerker, D.A., Ollinger, J.M., D'Esposito, M., 2004. Variation of BOLD hemodynamic responses across subjects and brain regions and their effects on statistical analyses. Neuroimage 21, 1639-1651.

Harvey, A.K., Pattinson, K.T., Brooks, J.C., Mayhew, S.D., Jenkinson, M., Wise, R.G., 2008. Brainstem functional magnetic resonance imaging: disentangling signal from physiological noise. J Magn Reson Imaging 28, 1337-1344.

Hayen, A., Herigstad, M., Wiech, K., Pattinson, K.T., 2015. Subjective evaluation of experimental dyspnoea--effects of isocapnia and repeated exposure. Respir Physiol Neurobiol 208, 21-28.

Kelly, M.E., Rowland, M.J., Okell, T.W., Chappell, M.A., Corkill, R., Kerr, R.S., Westbrook, J., Jezzard, P., Pattinson, K.T., 2013. Pseudo-continuous arterial spin labelling MRI for non-invasive, whole-brain, serial quantification of cerebral blood flow following aneurysmal subarachnoid haemorrhage. Transl Stroke Res 4, 710-718.

Luchtmann, M., Jachau, K., Tempelmann, C., Bernarding, J., 2010. Alcohol induced region-dependent alterations of hemodynamic response: implications for the statistical interpretation of pharmacological fMRI studies. Exp Brain Res 204, 1-10.

MacIntosh, B.J., Pattinson, K.T., Gallichan, D., Ahmad, I., Miller, K.L., Feinberg, D.A., Wise, R.G., Jezzard, P., 2008. Measuring the effects of remifentanil on cerebral blood flow and arterial arrival time using 3D GRASE MRI with pulsed arterial spin labelling. J Cereb Blood Flow Metab 28, 1514-1522.

Mitsis, G.D., Governo, R.J., Rogers, R., Pattinson, K.T., 2009. The effect of remifentanil on respiratory variability, evaluated with dynamic modeling. J Appl Physiol (1985) 106, 1038-1049.

Muthukumaraswamy, S.D., Evans, C.J., Edden, R.A., Wise, R.G., Singh, K.D., 2012. Individual variability in the shape and amplitude of the BOLD-HRF correlates with endogenous GABAergic inhibition. Hum Brain Mapp 33, 455-465.

Okell, T.W., Chappell, M.A., Kelly, M.E., Jezzard, P., 2013. Cerebral blood flow quantification using vessel-encoded arterial spin labeling. J Cereb Blood Flow Metab 33, 1716-1724.

Pattinson, K.T., Governo, R.J., MacIntosh, B.J., Russell, E.C., Corfield, D.R., Tracey, I., Wise, R.G., 2009. Opioids depress cortical centers responsible for the volitional control of respiration. J Neurosci 29, 8177-8186.

Wise, R.G., Pattinson, K.T., Bulte, D.P., Chiarelli, P.A., Mayhew, S.D., Balanos, G.M., O'Connor, D.F., Pragnell, T.R., Robbins, P.A., Tracey, I., Jezzard, P., 2007. Dynamic forcing of end-tidal carbon dioxide and oxygen applied to functional magnetic resonance imaging. J Cereb Blood Flow Metab 27, 1521-1532.
